# Effects of Invasive Winter Moth Defoliation on Tree Radial Growth in Eastern Massachusetts, USA

**DOI:** 10.3390/insects5020301

**Published:** 2014-04-09

**Authors:** Michael J. Simmons, Thomas D. Lee, Mark J. Ducey, Joseph S. Elkinton, George H. Boettner, Kevin J. Dodds

**Affiliations:** 1 Department of Natural Resources and the Environment, University of New Hampshire, 114 James Hall, Durham, NH 03824, USA; E-Mails: tom.lee@unh.edu (T.D.L.); mark.ducey@unh.edu (M.J.D.); 2 Department of Environmental Conservation, University of Massachusetts, 310 Agricultural Engineering Building, Amherst, MA 01003, USA; E-Mails: elkinton@ent.umass.edu (J.S.E.); boettner@psis.umass.edu (G.H.B.); 3 USDA Forest Service, Northeastern Area State & Private Forestry, 271 Mast Road, Durham, NH 03824, USA; E-Mail: kdodds@fs.fed.us

**Keywords:** *Operophtera brumata*, exotic species, dendroecology, *Quercus rubra*

## Abstract

Winter moth, *Operophtera brumata* L. (Lepidoptera: Geometridae), has been defoliating hardwood trees in eastern Massachusetts since the 1990s. Native to Europe, winter moth has also been detected in Rhode Island, Connecticut, eastern Long Island (NY), New Hampshire, and Maine. Individual tree impacts of winter moth defoliation in New England are currently unknown. Using dendroecological techniques, this study related annual radial growth of individual host (*Quercus* spp. and *Acer* spp.) trees to detailed defoliation estimates. Winter moth defoliation was associated with up to a 47% reduction in annual radial growth of *Quercus* trees. Latewood production of *Quercus* was reduced by up to 67% in the same year as defoliation, while earlywood production was reduced by up to 24% in the year following defoliation. Winter moth defoliation was not a strong predictor of radial growth in *Acer* species. This study is the first to document impacts of novel invasions of winter moth into New England.

## 1. Introduction

Winter moth, *Operophtera brumata* L. (Lepidoptera: Geometridae), has been responsible for widespread defoliation of trees (e.g., 36,000 ha in 2011) in Massachusetts for over a decade [1]. Native to Europe, winter moth was identified in southeastern Massachusetts in 2003 and is believed to have been responsible for heavy defoliation since the 1990s [2]. The distribution of winter moth in Massachusetts is expanding and, since its initial detection in 2003, it has been detected throughout much of eastern Massachusetts and portions of Rhode Island, Connecticut, eastern Long Island (NY), New Hampshire, and Maine [2]. The biological extent of winter moth in New England is now comparable to that of the burgeoning gypsy moth, *Lymantria dispar* L. (Lepidoptera: Lymantriidae) population in the early 1900s [3,4]. In portions of its range in New England where it is well-established, winter moth may now dominate the spring defoliator guild that includes fall cankerworm, *Alsophila pometaria* Harris (Lepidoptera: Geometridae), Bruce spanworm, *O. bruceata* Hulst (Lepidoptera: Geometridae), forest tent caterpillar, *Malacosoma disstria* Hübner (Lepidoptera: Lasiocampidae), and gypsy moth.

Winter moth is an important and well-studied defoliator in European forests [5,6,7]. While recently discovered in Massachusetts, previous invasions of winter moth have occurred in North America, with introductions in Nova Scotia in the early 1930s [8] and the Pacific Northwest in the 1950s [9,10]. Winter moth feeds on a variety of hardwood trees and shrubs in its native and exotic ranges and can be a serious pest of orchards, landscape trees, and deciduous forests [8,11,12]. In Nova Scotia, winter moth defoliates *Malus* spp., *Quercus rubra* L., *Ulmus americana* L., *Acer rubrum* L., *Tilia americana* L., and *Ostrya virginiana* (Mill.) K. Koch, as well as other deciduous tree species [13]. In Massachusetts, heavy winter moth defoliation has been observed (by the current authors) on several tree species including *Quercus*, *Acer*, and *Betula* species, among others, however the impact of this novel defoliation to radial growth has not yet been documented in the primary literature.

Winter moth is an early-season defoliator that feeds in the expanding buds and leaves of its hardwood host [6]. The life history of winter moth in New England is similar to that in Nova Scotia and Europe. Briefly, winter moth is a univoltine species that overwinters as an egg; larval eclosion typically occurs in April and larvae feed in the expanding buds and later on the foliage for approximately six weeks, with much of the damage often occurring inside the buds prior to leaf expansion. Pupation occurs in the soil beginning in mid- to late May. Adults emerge from the soil in late fall to early winter, when, upon mating, the flightless female lays eggs in bark crevices and on branches of selected host trees [13,14]. Host preference and the level of defoliation associated with winter moth larval feeding is largely determined by the degree of synchrony between budburst and larval eclosion [6,15,16].

The effects of winter moth defoliation on host tree radial growth have been studied in its native and exotic ranges. In England, Varley and Gradwell [5] found a radial growth reduction in *Quercus robur* L. trees defoliated by winter moth and the green oak leaf roller, *Tortrix viridana* L. (Lepidoptera: Tortricidae). Tikkanen and Roininen [7] found a radial growth reduction in *Prunus padus* L., *Sorbus aucuparia* L., and *Populus tremula* L. trees defoliated by winter moth in eastern Fennoscandia. In Nova Scotia, where a prolonged outbreak was eventually controlled by the release of biological control agents [11], winter moth defoliation reduced radial growth and basal area of *Q. rubra* [17], and repeated defoliation caused decline and mortality of this species [13]. This study is the first to document the radial growth impacts of the novel invasion of winter moth in New England and builds on the observations of Varley and Gradwell [5] in which the authors found a negative relationship between *Q. robur* latewood growth and the combined density of winter moth and the green oak leaf roller. Whereas Varley and Gradwell [5] indirectly related insect defoliation—using insect density as a surrogate—to intra-annual (*i.e.*, earlywood and latewood) variation in radial growth, our study directly relates rigorous estimates of actual winter moth defoliation to total, earlywood, and latewood radial growth of *Quercus* spp. and *Acer* spp. trees.

Dendroecology, the analysis of annual wood rings, is an effective tool used in the study of biological invasions [18,19] and dendroecological techniques have been used to elucidate the effects of insect defoliation on tree radial growth [20]. Measuring the effect of defoliation on radial growth of host trees is a logical step when gauging the potential impacts of novel invasions of forest defoliators, as radial growth can be used as a predictor of tree mortality [21,22]. 

Understanding the influence of winter moth defoliation intensity on tree growth in Massachusetts will provide important information on the threat of winter moth invasions in New England hardwood forests, while also providing guidance to pest management strategies. The objective of this study was to estimate the impact of winter moth defoliation intensity on the radial growth of defoliated host trees using analysis of tree cores. To meet this objective we took advantage of defoliation data collected from a previous study. Beginning in 2004 [23], individual trees were selected to document the population ecology of winter moth throughout its burgeoning outbreak in Massachusetts. Among the data collected were estimates of defoliation on specific trees attributable to winter moth and the availability of these data allowed the present study to proceed. In 2011, tree cores were extracted from the individual trees from which defoliation estimates were collected and radial growth patterns of these cores were related to these annual estimates of winter moth defoliation.

## 2. Methods

### 2.1. Winter Moth Defoliation Sampling

Eastern Massachusetts is part of the Northeastern Coastal Zone ecoregion [24] and comprises the southern New England Coastal Plain, Bristol Lowlands, and Boston Basin ecological subregions [24,25]. Eastern Massachusetts is defined by a mosaic of urban areas and State and private forests dominated by *Quercus*, *Acer*, and *Pinus* [24]. Soils in this region are predominantly Inceptisols and Entisols [25] formed mostly on acidic sedimentary, acidic granitic, and mafic/intermediate granitic bedrock [24]. 

Annual levels of winter moth defoliation were quantified (percent leaf area removed by larval feeding) from 2004–2010 on seven long-term winter moth study trees that spanned the area infested by winter moth in eastern Massachusetts and included *Q. rubra*, *Q. velutina* L., *A. rubrum*, and *A. saccharum* Marsh (Table 1). The locations of the trees used for this study varied from private house lots with individual open-grown trees, to trees at the edge of forested tracts. Sites were selected by University of Massachusetts, Amherst (UMass) researchers in 2004, following the 2003 confirmation of winter moth in Massachusetts. Study trees were selected so as to (1) be spread out across the area infested by winter moth; (2) include *A. rubrum*, *Q. rubra* or *Q. velutina*; and (3) have no prospect of pesticide application to control winter moth or any other defoliator. To our knowledge, trees received no supplemental water nor were they fertilized during the sample period. Trees were selected based on ease of access to crowns to facilitate successful winter moth life stage (including egg and larval counts) and defoliation sampling (access for pole pruners). Although tree sampling began in 2004, more trees (at other locations) were added as winter moth spread into new areas. 

**Table 1:** Location, habitat (open grown, forest, forest edge), species, diameter at breast height (DBH; 1.4 m), age, years of winter moth defoliation estimates of “defoliation trees” and the number of ring width index (RWI) values (calculated using detrending method; see methods Section 2.3) with corresponding percent defoliation estimates in eastern Massachusetts, USA. Number of RWI values *vs.* % defoliation estimates determined by subtracting the first year of defoliation estimates per tree in order to match models evaluating previous year defoliation estimates (see Methods section for details).

Tree #	Location	Habitat	Species	DBH (cm)	Age	Years w/Defoliation Estimates	# RWI *vs.* % Defoliation
*Quercus* 1	Hanson, MA, USA	Open	*Quercus velutina*	25.6	15	2004–2010	6
*Acer* 1		Open	*Acer rubrum*	53.1	71	2004–2010	6
*Quercus* 2	Hanson, MA, USA	Edge	*Quercus rubra*	37.6	58	2004–2010	6
*Quercus* 3	Hingham, MA, USA	Open	*Quercus rubra*	35.1	28	2004–2010	6
*Acer* 2		Forest	*Acer rubrum*	24.8	38	2004–2010	6
*Quercus* 4	Hingham, MA, USA	Open	*Quercus rubra*	29.1	44	2004–2010	6
*Acer* 3		Edge	*Acer rubrum*	34.2	43	2004–2010	6
*Quercus* 5	Wellesley, MA, USA	Edge	*Quercus velutina*	22.6	50	2008–2010	2
*Acer* 4		Edge	*Acer saccharum*	19.5	45	2009–2010	1
*Quercus* 6	Wenham, MA, USA	Open	*Quercus velutina*	30.8	21	2006–2010	4
*Acer* 5		Open	*Acer rubrum*	34.4	34	2006–2010	4
*Quercus* 7	West Bridgewater, MA, USA	Open	*Quercus rubra*	37.6	27	2004–2005	1
*Quercus* 8		Open	*Quercus rubra*	40.7	22	2004–2010	6
*Acer* 6		Edge	*Acer rubrum*	26.7	45	2004–2010	6

Each year in late May or early June when winter moth feeding had finished and the larvae had pupated, pole pruners were used to cut leaf clusters from throughout the canopy of each sample tree. An effort was made to sample leaves throughout the canopy to capture the overall level of defoliation. Thirty leaves were selected from the leaf clusters and these were dried and pressed for later analysis. Percent defoliation was estimated by visually rating each leaf on a 10-class scale (0–10%, 11%–20% ... 91%–100%) and averages were computed for the 30 leaves from each tree. Estimates of leaf re-flushing were not included in the study. Except in extreme cases in which winter moth defoliation results in 100% removal of the leaf, winter moth larvae often consume a varying percentage of the leaf tissue. Thus, percentage of leaf lost to defoliation is an appropriate metric for winter moth defoliation, as opposed to overall leaf area reduction as has been used in studies of other defoliators, e.g., [26]. While other defoliators, including gypsy moth and forest tent caterpillar, were present in the region during the early years (2004–2006) of the population ecology study, these defoliators were never abundant on the sample trees and winter moth was invariably the most abundant defoliator during this time period.

### 2.2. Increment Core Collection

There were three categories of trees selected for radial growth analysis, (1) defoliation trees; (2) cross-dating trees and; (3) control trees. Defoliation trees were the same trees on which defoliation levels were previously estimated and included five *Q. rubra*, three *Q. velutina*, six *A. rubrum* and one *A. saccharum* trees. Two increment cores, separated by at least 60°, were extracted from each sample tree at breast height (1.4 m) using increment borers; we were unable to extract a viable core from *Quercus* defoliation tree number 3; thus cores for *Quercus* 3 (Table 1) were extracted from a *Q. rubra* growing directly adjacent to the tree on which defoliation estimates were collected. Location of core extraction was based on tree bole orientation and growth form. In addition, to assist in subsequent cross-dating of cores, each study tree was paired with a nearby tree of the same species from which two cores were also extracted (except for one site, West Bridgewater, which contained two *Q. rubra* defoliation trees and no other *Quercus* trees; Table 1). These “cross-dating trees” were selected to increase the number of trees used in cross-dating of radial growth trends. 

We also cored *Pinus strobus* L. trees as a “control species”, one not fed upon by winter moth. We expected that ring widths of the control species would not vary with winter moth defoliation of host species. Occurrence of ring width reduction in both host and non-host (control) species, however, would suggest the existence of extraordinary environmental variables correlated with winter moth activity, weakening the inference that winter moth alone caused the reduction. In contrast, ring width reduction in host but not control species would strengthen the inference that winter moth was the causal variable. For these control trees, two cores were extracted from two *P. strobus* trees (herein referred to as *Pinus*) neighboring each “defoliation tree”; except at one location, West Bridgewater, in which *Pinus* trees were absent. 

Tree cores were labeled in the field and returned to the lab for preparation and analysis. In total (including defoliation trees, cross-dating trees, and control trees), two increment cores per tree were sampled from 14 *Quercus* trees, 14 *Acer* trees, and 12 *Pinus* trees from throughout eastern Massachusetts.

### 2.3. Core Preparation and Analysis

Increment cores were stored in paper straws and allowed to dry. Cores were then glued to wooden mounts and sanded with progressively finer sandpaper, concluding with a 600-grit. A Velmex measurement system (Velmex, East Bloomfield, NY, USA), in combination with Measure J2X v. 4.1.2 software (VoorTech Consulting, Holderness, NH, USA) was used to measure annual growth ring widths to the nearest 0.001 mm. Once measured, cores were visually cross-dated based on event years (*i.e.*, years having low growth due to known drought, insect defoliation, *etc.*; [27,28]) using scatterplots created in MS Excel. Cores were cross-dated by species using each of the two core series per tree for the defoliation trees and cross-dating trees combined. During cross-dating, the cores from two *A. rubrum* trees (including one defoliation tree and one cross-dating tree from the same site) were removed due to rot or unreadable growth rings. The program COFECHA [29] was used to verify cross-dating and to assess measurement error. A Pearson critical correlation level of 0.328 (99% confidence level) was used as a metric of cross-dating accuracy in which individual cores were correlated with the master chronology of the respective species [29]. Individual cores that fell below this level were double-checked and re-measured in an effort to insure measurement accuracy; however, an individual correlation with the master chronology below 0.328 did not necessarily preclude the core from further analysis if the measurement was deemed accurate.

Once the entire collection of cores was cross-dated, the cross-dating trees were precluded from further analyses and the *Q. rubra* (*n* = 5), *Q. velutina* (*n* = 3), *A. rubrum* (*n* = 5) and *A. saccharum* (*n* = 1) defoliation trees (trees with quantified defoliation percentages) were used to evaluate defoliation impacts on tree radial growth. The *Pinus* control trees (*n* = 12) were used to support winter moth as a contributing factor to any radial growth trends displayed by host trees. If winter moth defoliation was merely correlated with some other extraordinary physical variable that affected host species ring width, we would also expect to see a similar response in *Pinus* radial growth. Thus, we regressed *Pinus* radial growth on the defoliation estimates from neighboring defoliation trees. *Quercus rubra* and *Q. velutina* belong to the red oak group (*Erythrobalanus*) and readily hybridize [30]; these species were combined for analysis and are herein referred to as *Quercus*. *Acer saccharum* was combined with *A. rubrum* for analysis, and are herein referred to as *Acer*. Therefore, a total of eight *Quercus*, six *Acer*, and 12 *Pinus* trees were used to analyze defoliation impacts. The number of trees used to estimate defoliation effects is small, a consequence of the cost in time and labor of obtaining high quality estimates of defoliation. However, as each tree was examined over multiple years the actual number of ring width observations is high (see below).

In order to remove age-related growth trends in ring width, the raw ring width data of the study trees were detrended and converted to a dimensionless ring-width index (RWI) following procedures outlined by Bunn [31]. Using the dplR computer package [31] within the R statistical program [32], a modified negative exponential curve was fit to the raw ring width data for each core; subsequently, these data were detrended within dplR by dividing the annual raw ring widths by the predicted values estimated by the fitted modified negative exponential curve. The detrended data for the two cores per tree were averaged and provided one average RWI per year per study tree. 

In addition to total annual ring widths, earlywood increment was measured for the *Quercus* study trees in years of known defoliation levels. *Quercus* are ring-porous tree species; thus, earlywood and latewood were identified based on changes in vessel size and wood color [26]. Proportions of each ring that were earlywood and latewood were calculated. Detrended earlywood and latewood ring width indices (RWI) were calculated by multiplying the respective proportions by the total RWI. 

### 2.4. Statistical Analysis

Statistical analyses were performed using the JMP 9.0 software (SAS, Cary, NC, USA). Using simple linear regression, the ring width index (RWI) of each tree species group was regressed on tree age to evaluate the effectiveness of the detrending technique; the correlation between the two variables was assessed by the adjusted r^2^ (r^2^*_adj_*) and *p*-values. This revealed no relationship between tree age and RWI, thereby supporting the detrending technique. 

Multicollinearity of regressors was tested using variance inflation factors (VIF). Studentized Residuals were calculated to check for the presence of outliers and to test the normality of the response variables. In addition, Cook’s D values were calculated to test for influential observations. Subsequently, *Acer* RWI and *Pinus* RWI were normalized using a 10 × log_10_ transformation. In addition, the proportions of *Quercus* earlywood and latewood were normalized using the arcsine square root transformation. An influential observation was noted in the *Acer* RWI data; this point was checked and determined to be a valid observation. Statistical analyses performed with, and without, the influential observation revealed no change in the results. Thus, the observation in question was retained. 

Multiple regression was used to determine which variables had the greatest effect on ring width index (RWI) for *Quercus* and *Acer*; for *Pinus*, we matched the best model for *Quercus* RWI. We hypothesized that winter moth defoliation in the current year, the previous year, and the combination of the two years would negatively influence host tree radial growth. Model selection for *Quercus* and *Acer* RWI was performed using the corrected Akaike Information Criterion (AIC*c*) value [33], wherein the model with the lowest AIC*c* value was considered the “best” model in the set of models run for each response variable. However, models with AIC*c* simple differences (ΔAIC*c*) values less than or equal to two were considered highly competitive models and were retained for discussion. 

The variables *tree* and *year* were considered nominal random variables and were entered into candidate models as (random effects) covariates to account for random variation due to individual tree and site characteristics (*tree*) and climate (*year*). *Percent current year winter moth defoliation* was treated as a fixed effect and was defined as the percentage of leaf surface lost by winter moth defoliation during a given growing season. For example, the percent winter moth defoliation in the year 2008 would correspond with the radial growth increment of 2008. *Percent previous year winter moth defoliation* was also treated as a fixed effect and was defined as the percentage of leaf surface lost by winter moth defoliation of a given tree during the previous year growing season. For example, the previous year defoliation for the 2008 radial growth increment would be defined as the 2007 winter moth defoliation percentage. The effect of the interaction between *percent current year winter moth defoliation* and *percent previous year winter moth defoliation* was also included in the model; this interaction was treated as a fixed effect. Defoliation data were available beginning in 2004. Thus, previous year defoliation levels were only available from 2005 and onwards. The application of AIC*c* for model selection requires that identical datasets be used in all models compared. As such, the year 2004 was excluded from the model analyses. Similarly, for trees added after 2004 (as winter moth moved into new areas), the first year of defoliation estimates was excluded.

For analysis, estimates of defoliation were pooled by species. Given that we accounted for *year* and *tree* (as random effects), the number of observations for our multiple regression analyses were not restricted to the number of “defoliation trees”. Rather, the number of observations per species was determined by the number of defoliation trees *multiplied by* the number of years in which defoliation estimates were made (again, minus the first year of defoliation to account for previous year defoliation estimates). For example, *Quercus* 1 (Table 1) had defoliation estimates from 2004–2010; when removing the year 2004, this equates to six years of defoliation estimates with corresponding RWI values. Thus, the number of observations included 37 RWI values for *Quercus*, 29 RWI values for *Acer*, and 60 RWI values for *Pinus*.

Multiple regression was also used to determine the role of *percent current year winter moth defoliation*, *percent previous year winter moth defoliation*, the interaction of current year and previous year winter moth defoliation, *tree*, and *year* in explaining *Quercus* earlywood (EW) and latewood (LW) RWI. As before, the model with the lowest AIC*c* value was adopted as the best model. In addition, the proportions of earlywood and latewood were separately regressed on total *Quercus* RWI to determine their relationships with total annual ring width index. 

We tested for serial correlation in the response variable in each of our “best” models using the Durbin-Watson test. If random effects were retained in the model, which precluded the use of the Durbin-Watson test, we used two methods to test for serial correlation. First, we assessed serial correlation between the model residuals and the one-year lagged residuals using an ordinary Pearson correlation coefficient. Second, we dropped the random effect from the model and tested for serial correlation once again using a Durbin-Watson test.

## 3. Results

The *Quercus* (*n* = 8) used in defoliation analyses had a mean diameter of 32.4 (SE ± 2.3) cm and a mean age of 33 (SE ± 5.3) years. *Acer* (*n* = 6) had a mean diameter of 32.1 (SE ± 4.8) cm and an average age of 45 (SE ± 5.7) years. *Pinus* (*n* = 12) had a mean diameter of 34.0 (SE ± 3.3) cm and a mean age of 50 (SE ± 4.9) years (Table 1). Observed levels of defoliation ranged from 4% to 95% for *Quercus* and from 0% to 52% for *Acer*. 

A total of 16 models were evaluated for each response variable (*Quercus* RWI, *Quercus* EW RWI, *Quercus* LW RWI, *Acer* RWI, and *Pinus* RWI). These models included the global model (*percent current year winter moth defoliation*, *percent previous year winter moth defoliation*, the interaction of current year and previous year winter moth defoliation, *tree*, and *year*) and each nested iteration. 

**Figure 1:**
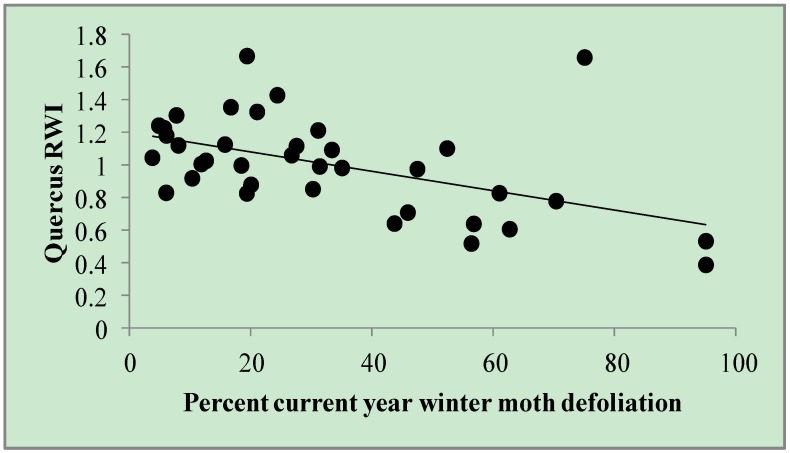
*Quercus* RWI by *percent current year winter moth defoliation*. Defoliation estimates (*n* = 37) among eight trees throughout eastern Massachusetts from 2005–2010. *Quercus* RWI = 1.202 – 0.006 × (*% current year winter moth defoliation*). r^2^*_adj_* = 0.24.

For *Quercus* RWI, one model was retained (Table 2). Based on this model (ΔAIC*c* = 0.0; r^2^*_adj_* = 0.24), variation in *Quercus* RWI was most strongly explained by *percent current year winter moth defoliation* (parameter estimate = −0.006; SE ± 0.0017), wherein winter moth defoliation was associated with up to a 47% reduction in *Quercus* RWI (Figure 1). There was no serial correlation in the residuals from this model (Durbin-Watson = 1.899; auto correlation = 0.0019; *p* = 0.3753).

**Table 2:** Results of multiple regression with *Quercus*, *Acer*, and *Pinus* response variables from the years 2005–2010 with defoliation estimates on individual trees throughout eastern Massachusetts. Parameter estimates (PE) are shown for fixed effects, variance components (VC) are provided for random effects. RWI = ring width index; EW = earlywood; LW = latewood; SE = standard error; ΔAIC*c* = corrected Akaike Information Criterion simple differences.

						Log	Akaike	Evidence
Model	Model Effects	ΔAIC*c*	r^2 adj	PE/VC	SE	Likelihood	Weight	Ratio
***Quercus* RWI**								
	% current year	0.00	0.24	−0.006	0.002	1.00	0.72	1.00
***Quercus* EW RWI**								
	% previous year	0.00	0.77	−0.001	0.000	1.00	0.68	1.00
	tree (random)			0.010	0.006			
***Quercus* LW RWI**								
	% current year	0.00	0.35	−0.006	0.001	1.00	0.70	1.00
***Acer* RWI**								
	% previous year	1.41	−0.02	−0.002	0.003	0.49	0.13	2.02
	% previous year	1.02	0.72	0.000	0.002	0.60	0.16	1.67
	tree (random)			0.042	0.030			
	% current year	0.09	0.02	−0.004	0.003	0.96	0.25	1.05
	% current year	0.00	0.73	−0.002	0.002	1.00	0.26	1.00
	tree (random)			0.041	0.029			
***Pinus* RWI**								
	% current year	0.00	0.02	0.002	0.001	1.00	0.43	1.00

Analysis of the *Quercus* earlywood and latewood growth indices revealed the complexity behind the total *Quercus* RWI reduction. Based on the best model (ΔAIC*c* = 0.0; r^2^*_adj_* = 0.77; Table 2), *Quercus* earlywood RWI was negatively related to *percent previous year winter moth defoliation* (parameter estimate = −0.001; SE ± 0.0005) and the random effect of *tree* (variance component = 0.01; SE ± 0.006). This relationship resulted in up to a 24% reduction in *Quercus* earlywood RWI. There was strong serial correlation in the residuals of this model (Pearson Correlation = 0.7895; *p* < 0.001). Consequently, the confidence limits on the model might not be as conservative as they would be in the absence of serial correlation and the AIC*c* evaluation may include marginal variables in the model. When the random effect of tree was removed from the model, there was no correlation between the residuals of *Quercus* earlywood RWI *vs.* % previous year defoliation (Durbin-Watson = 2.122; auto correlation = −0.07023; *p* = 0.6393). 

**Figure 2:**
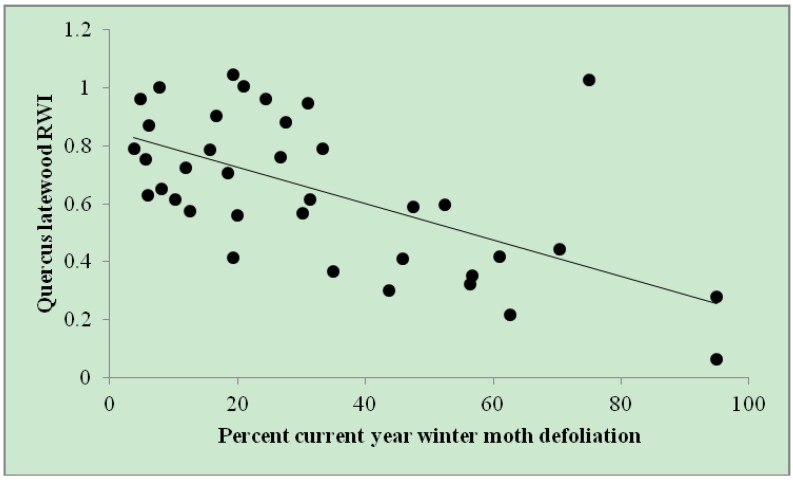
*Quercus* latewood RWI by *percent current year winter moth defoliation*. Defoliation estimates (*n* = 37) among eight trees throughout eastern Massachusetts from 2005–2010. *Quercus* LW RWI = 0.851 – 0.006 × (*% current year winter moth defoliation*). r^2^*_adj_* = 0.35.

*Quercus* latewood RWI was negatively related to *percent current year winter moth defoliation* (parameter estimate = −0.006; SE ± 0.001), based on the one retained model (ΔAIC*c* = 0.00; r^2^*_adj_* = 0.35; Table 2). This relationship resulted in up to a 67% reduction in *Quercus* latewood RWI (Figure 2). There was no serial correlation in the residuals from this model (Durbin-Watson = 2.025; auto correlation = −0.0608; *p* = 0.5279). 

**Figure 3:**
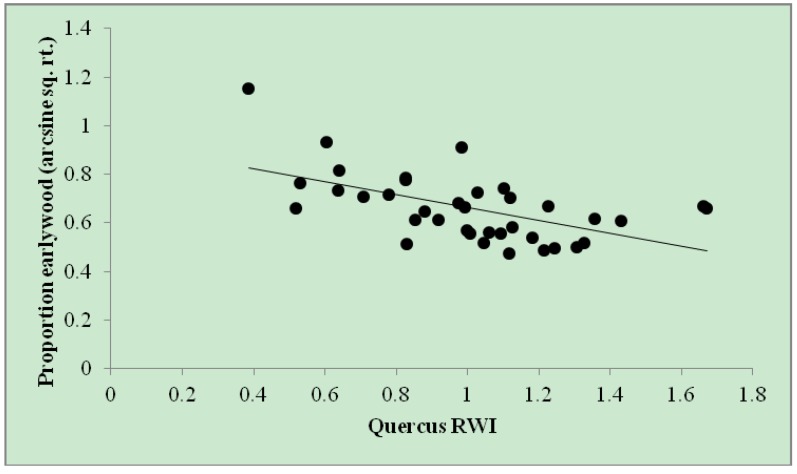
Proportion of *Quercus* earlywood by *Quercus* RWI on annual ring widths (*n* = 37) among eight trees throughout eastern Massachusetts from 2005–2010. Proportion earlywood (arcsine sq. rt.) = 0.929 – 0.267 × (*Quercus* RWI). r^2^*_adj_* = 0.29.

Simple regression analysis found that the proportion of earlywood in a given year decreased (and, thus, the proportion of latewood increased) with total *Quercus* RWI (r^2^*_adj_* = 0.29; Figure 3). This relationship indicates that increased *Quercus* RWI drives a reduction in the proportion of earlywood and an increase in latewood proprotion and lends further support for the *Quercus* RWI model wherein *percent current year winter moth defoliation* was the strongest predictor of *Quercus* RWI, as *percent current year winter moth defoliation* was shown (above) to reduce *Quercus* LW RWI. 

For *Acer* RWI, the best model (ΔAIC*c* = 0.00; r^2^*_adj_* = 0.73; Table 2) contained *percent current year winter moth defoliation* (parameter estimate = −0.002; SE ± 0.002) and *tree* (variance component = 0.04; SE ± 0.03). In addition to the best model, there were three competing models (ΔAIC*c* = 0.09 to 1.41) that attempted to explain *Acer* RWI. Based on the r^2^*_adj_* values of these competing models (Table 2), the majority of the variation in *Acer* RWI was explained by *tree* rather than winter moth defoliation. There was strong serial correlation in the residuals of the best *Acer* RWI model (Pearson Correlation = 0.7001; *p* = 0.0002). Consequently, the confidence limits on this model might not be as conservative as they would be in the absence of serial correlation and the AIC*c* evaluation may include marginal variables in the model. When the random effect of tree was removed from the model, there was no correlation between the residuals of *Acer* RWI *vs.* % current year winter moth defoliation (Durbin-Watson = 2.036; auto correlation = −0.0606; *p* = 0.5038).

There was no relationship (r^2^*_adj_* = 0.02; Table 2) between non-host (control) *Pinus* RWI and winter moth defoliation when matched with the best model for *Quercus* RWI [*percent current year winter moth defoliation* (parameter estimate 0.002; SE ± 0.001) as the sole explanatory variable]. 

## 4. Discussion

Winter moth is well-established in southern New England [2] and has been responsible for extensive tree defoliation throughout eastern Massachusetts. The forests of northeastern USA have a long history of invasion by exotic species [34]. European gypsy moth, hemlock woolly adelgid, *Adelges tsugae* Annand (Homoptera: Adelgidae), Asian longhorned beetle, *Anoplophora glabripennis* Motschulsky (Coleoptera: Cerambycidae), Dutch elm disease, *Ophiostoma novo-ulmi* Brasier and *Ophiostoma ulmi* (Buisman) Melin and Nannf.), chestnut blight, *Cryphonectria parasitica* (Murrill) M.E. Barr), and beech bark disease complex have all dramatically altered, or threaten to alter, native forest ecosystems of this region [18,35,36,37,38]. Our results suggest that invasion by winter moth may act as an additional stressor in these forests. Although our study focused on individual trees growing in the open or in forest edges, our results may provide an estimate on the impact of winter moth defoliation on forest trees. However, if applied to a forested situation, the results presented here are likely conservative, as the influence of competition with neighboring trees was reduced in this study. 

Winter moth defoliation reduced the annual radial growth of infested *Quercus* trees in eastern Massachusetts by up to 47% in the year of defoliation. That this reduction is due to winter moth defoliation and not some stochastic change in the physical environment is suggested by the fact that winter moth defoliation failed to explain much variation in *Pinus* radial growth. Moreover, the positive parameter estimate for *percent current year winter moth defoliation* (parameter estimate = 0.002, Table 2) in the best model for *Pinus* RWI suggests that winter moth defoliation on neighboring host trees may have a slight release effect on *Pinus* radial growth. Thus, the radial growth reduction shown by *Quercus* cannot likely be explained by environmental factors that happen to correlate with winter moth defoliation levels. 

The large amount of variation in *Acer* RWI that was explained by *tree* may have been caused by great variation in *Acer* ring widths among trees (including the defoliation and cross-dating trees). The series inter-correlation for the *Acer* tree cores (0.055) was well below the critical correlation level (0.328) used in COFECHA. Numerous efforts were made to ensure the measurement accuracy of these cores. Consequently, the low series inter-correlation was likely a result of natural variation in *Acer* growth across our sites. The low series inter-correlation value limits any discussion of the effects of winter moth defoliation on *Acer* radial growth. However, Heichel and Turner [39] found that defoliated *A. rubrum* has a higher rate of CO_2_ assimilation in residual primary and regrowth foliage than *Q. rubra*. Moreover, *A. rubrum* branch growth and leaf production may be less severely affected by defoliation than *Q. rubra* [40]. Although not evaluated in our study, this comparative resilience of *A. rubrum* to defoliation may partially explain the lack of response of *Acer* RWI to winter moth defoliation that we observed. 

Although we accounted for random variation due to individual tree, site characteristics (e.g., edaphic), and climate in our regression models it is possible that moisture or temperature extremes during our study period influenced the observed variation in RWI. In addition, it is also possible that other defoliators that were not observed influenced radial growth of our study trees. These and other potentially influential factors may account for residual variation in our regression models.

The presence of strong serial correlation in the “best” *Quercus* earlwood RWI (explanatory variables included *% previous year winter moth defoliation* and *tree*) model suggest that, for some *Quercus* trees, earlywood growth from one year to the next is highly correlated, which could indicate some environmental signal that influences more than one year of radial growth. As we included percent previous year and percent current year winter moth defoliation in the original models, this defoliation was factored out. Thus, the residual variation in earlywood RWI may be explained by some strong multi-year signal.

The effect of winter moth defoliation on *Quercus* radial growth was likely caused by a reduction in photosynthate production and/or allocation to radial growth, as increased levels of defoliation likely reduce the net CO_2_ assimilation within defoliated *A. rubrum* and *Q. rubra* [39] and repeated defoliation can reduce root carbon reserves in mature trees [41]. The earlywood and latewood growth reductions observed in our study are consistent with the phenology of winter moth and *Quercus*. As ring-porous species, the earlywood growth of *Quercus* is proportionally driven by resources produced and stored during previous growing seasons [42] and is initiated prior to spring bud burst (leaf expansion) [43,44]. Conversely, latewood growth of ring-porous species is mainly a function of photosynthate allocation in the year concurrent with growth [5,42]. Winter moth larval feeding occurs in the expanding buds in the early spring and is, therefore, concurrent with earlywood production. As such, winter moth defoliation in a given year does not have an effect on the earlywood growth for that year. However, as winter moth defoliates *Quercus* while the leaves are expanding, and since latewood growth is proportionally dependent upon actively produced photosynthate from within the current year, the photosynthetic capabilities of the defoliated tree may be reduced and/or photosynthate may be allocated to processes (e.g., defense, re-leafing) other than latewood growth. This relationship between defoliation in the current year and latewood growth has been demonstrated with other defoliators [26,45,46,47,48]. 

Although not quantified in this study, we have observed tree refoliation (*i.e.*, flushing of a second set of leaves) during heavy winter moth defoliation. This phenomenon may have influenced our results, accounting for variation in *Quercus* RWI and contributing to our lack of relationship between *Acer* and winter moth defoliation. Defoliation can induce compensatory production of photosynthate in the remaining or regrowth leaf tissue of trees [39,49,50]. However, increased levels of defoliation likely reduce the net CO_2_ assimilation within defoliated *A. rubrum* and *Q. rubra* [39]. Moreover, as repeated defoliation can reduce root carbon reserves in mature trees [41], the benefit of compensatory photosynthesis and/or refoliation is likely diminished after multiple years of defoliation. 

Our dendroecological analyses evaluated multiple aspects of tree radial growth and timing of defoliation over multiple years to expand the understanding of how winter moth impacts individual tree growth. Our results are consistent with, synthesize, and expand on previous studies that have evaluated the effects of winter moth defoliation in Nova Scotia and Europe. Embree [17] showed a reduction in radial growth and basal area of *Q. rubra* trees with increasing levels of winter moth defoliation in Nova Scotia. As part of their classic studies at Wytham Wood, England, Varley and Gradwell [5] found a negative relationship between the percent latewood growth of *Q. robur* and caterpillar densities of winter moth and the green oak leaf roller. The authors also suggested that high caterpillar densities may negatively influence earlywood growth in the same year as well [5]. While these previous studies focused on various aspects of defoliation and tree growth, we were able to investigate linkages between defoliation intensity, annual increment, and the two portions that comprise an annual ring (*i.e.*, earlywood and latewood). We observed a radial growth reduction of up to 67% in *Quercus* latewood RWI concurrent with increasing winter moth defoliation. Moreover, we found that *Quercus* earlywood RWI was also negatively influenced by winter moth defoliation (from the previous year) by up to 24%. This suggests that one year of winter moth defoliation may influence two years of radial growth. Our results further suggest that winter moth defoliation in New England may lead to tree decline and mortality, as repeated defoliation by winter moth caused crown dieback and tree mortality in *Q. rubra* stands in Nova Scotia [13,17]. Intensive stand sampling from sites throughout eastern Massachusetts showed that increased tree mortality is occurring in stands where winter moth has been present for multiple years [51]. 

The effect of winter moth defoliation on the radial growth of *Quercus* was similar to those documented for other outbreak species and further implies this insect species will have a negative effect on the growth and survivorship of *Quercus* trees in mixed forests of Massachusetts. Studies have found a negative relationship between defoliation by gypsy moth and the standardized total radial increment of *Q. rubra* trees [26,52]. Moreover, consistent with our results, Muzika and Liebhold [26] and Fajvan *et al.* [48] also noted a reduction in latewood growth of *Q. rubra* concomitant with gypsy moth defoliation. Further, earlywood production in *Quercus* species was reduced in the season following gypsy moth defoliation [48]. The implications of our findings are considerable, as gypsy moth is often referred to as one of the most devastating threats facing eastern U.S. forests [26,48] and these two species have potentially overlapping spatial and temporal distributions. The combined effects of these insects on tree growth and mortality are currently unknown, but could have serious consequences for tree health in northeastern forests as multiple defoliation events on deciduous trees in a given year may result in tree mortality [53].

In addition to radial growth reduction, insect defoliation can lead to widespread tree decline and mortality. In the northeastern United States, relationships between tree mortality and defoliation have been well-documented (e.g., [35,48,54,55,56]). Insect defoliation may initiate a chronosequence of decline and mortality that involves secondary wood borers and root decay fungi, notably *Armillaria* species [57,58]. Defoliating insects can alter forest stand dynamics [59] and exotic organisms may pose a serious threat to native forest ecosystems and forest management regimes [60,61]. The results presented here have relevance to novel introductions of winter moth into deciduous forests in eastern North America, where the species composition and climate offer ideal winter moth habitat [2,62]. 

Although the present study does not address the forest stand level impacts of winter moth defoliation, our results indicate that winter moth may influence tree mortality in *Quercus-*dominated forests in eastern Massachusetts. Radial growth decline can be used as a predictor of tree mortality [21,22] and multiple year winter moth defoliation events may contribute to mortality of *Q. rubra* [13,51]. Moreover, frequent defoliation (with periodicity ranging from 5 to 15 years) may reduce net ecosystem productivity [63]. Given the polyphagous feeding of winter moth it is difficult to infer species compositional changes in forests defoliated by winter moth. However, given the species composition of forests in our study region, the observed preferential feeding of winter moth on *Quercus*, and the possible lack of response of *Acer* to winter moth defoliation, suggest a shift from *Quercus-*dominated forests to increased importance of *Acer* and *Pinus*.

## 5. Conclusions

The results presented here indicate that winter moth is a significant threat to the forests of southern New England and *Quercus* forests throughout North America. As winter moth populations are well-established throughout eastern Massachusetts, and assuming the results presented herein can be extrapolated to other sites, the *Quercus* resource that dominates much of eastern Massachusetts will likely decline in the presence of winter moth defoliation. Given the growth decline associated with winter moth defoliation, current efforts to establish biological control agents [2] for this species are warranted.

## References

[1] USDA Forest Service Forest Health Protection and Massachusetts Department of Conservation and Recreation 2011 Massachusetts Forest Health Highlights. http://fhm.fs.fed.us/fhh/fhh_11/MA_FHH_2011.pdf.

[2] Elkinton J.S., Boettner G.H., Sremac M., Gwiazdowski R., Hunkins R.R., Callahan J., Scheufele S.B., Donahue C.P., Porter A.H., Khrimian A. (2010). Survey for winter moth (Lepidoptera: Geometridae) in northeastern North America with pheromone-baited traps and hybridization with the native Bruce spanworm (Lepidoptera: Geometridae). Ann. Entomol. Soc. Am..

[3] USDA Forest Service, F.H.T.E.T. National Forest Damage Agent Range Maps: Winter Moth. http://www.fs.fed.us/foresthealth/technology/fdar_maps.shtml/.

[4] USDA Forest Service Gypsy Moth in North America: An Atlas of Historical Gypsy Moth Defoliation and Quarantined Areas in the US. http://www.fs.fed.us/ne/morgantown/4557/gmoth/atlas/#spread/.

[5] Varley G.C., Gradwell G.R. The effect of partial defoliation by caterpillars on the timber production of oak trees in England. Proceedings of the 11th International Congress of Entomology.

[6] Feeny P. (1970). Seasonal changes in oak leaf tannins and nutrients as a cause of spring feeding by winter moth caterpillars. Ecology.

[7] Tikkanen O.P., Roininen H. (2001). Spatial pattern of outbreaks of *Operophtera brumata* in eastern Fennoscandia and their effects on radial growth of trees. For. Ecol. Manag..

[8] Embree D.G. (1965). The population dynamics of winter moth in Nova Scotia, 1954–1962. Mem. Entomol. Soc. Can..

[9] Gillespie D.R., Finlayson T., Tonks N.V., Ross D.A. (1978). Occurrence of the winter moth, *Operophtera brumata* (Lepidoptera: Geometridae), on southern Vancouver Island, British Columbia. Can. Entomol..

[10] Roland J. (1988). Decline in winter moth populations in North America—Direct *versus* indirect effect of introduced parasites. J. Anim Ecol..

[11] Embree D.G. (1991). The winter moth *Operophtera brumata* in eastern Canada, 1962–1988. For. Ecol. Manag..

[12] Wylie H.G. (1960). Insect parasites of the winter moth, *Operophtera brumata*, (L.) (Lepidoptera: Geometridae) in western Europe. Entomophaga.

[13] Cuming F.G. (1961). The distribution, life history, and economic importance of the winter moth, *Operophtera brumata* (L.) (Lepidoptera: Geometridae) in Nova Scotia. Can. Entomol..

[14] Varley G.C., Gradwell G.R., Hassell M.P. (1973). Insect Population Ecology: An Analytical Approach.

[15] Tikkanen O.P., Julkunen-Tiitto R. (2003). Phenological variation as protection against defoliating insects: The case of *Quercus robur* and *Operophtera brumata*. Oecologia.

[16] Tikkanen O.P., Lyytikainen-Saarenmaa P. (2002). Adaptation of a generalist moth, *Operophtera brumata*, to variable budburst phenology of host plants. Entomol. Exp. Appl..

[17] Embree D.G. (1967). Effects of winter moth on growth and mortality of red oak in Nova Scotia. For. Sci..

[18] Dodds K.J., Orwig D.A. (2011). An invasive urban forest pest invades natural environments—Asian longhorned beetle in northeastern US hardwood forests. Can. J. For. Res..

[19] Burnham K.M., Lee T.D. (2010). Canopy gaps facilitate establishment, growth, and reproduction of invasive *Frangula alnus* in a *Tsuga canadensis* dominated forest. Biol. Invasions.

[20] Fritts H.C., Swetnam T.W. (1989). Dendroecology—A tool for evaluating variations in past and present forest environments. Adv. Ecol. Res..

[21] Monserud R.A. (1976). Simulation of forest tree mortality. For. Sci..

[22] Wyckoff P.H., Clark J.S. (2000). Predicting tree mortality from diameter growth: A comparison of maximum likelihood and Bayesian approaches. Can. J. For. Res..

[23] Whited B. (2007). The Population Ecology of Winter Moth (Operophtera brumata) in Eastern Massachusetts.

[24] Hall B., Motzkin G., Foster D.R., Syfert M., Burk J. (2002). Three hundred years of forest and land-use change in Massachusetts, USA. J. Biogeogr..

[25] McDonald R.I., Motzkin G., Bank M.S., Kittredge D.B., Burk J., Foster D.R. (2006). Forest harvesting and land-use conversion over two decades in Massachusetts. For. Ecol. Manag..

[26] Muzika R.M., Liebhold A.M. (1999). Changes in radial increment of host and nonhost tree species with gypsy moth defoliation. Can. J. For. Res..

[27] Yamaguchi D.K. (1991). A simple method for cross-dating increment cores from living trees. Can. J. For. Res..

[28] Tardif J.C., Conciatori F. (2006). A comparison of ring-width and event-year chronologies derived from white oak (*Quercus alba*) and northern red oak (*Quercus rubra*), southwestern Quebec, Canada. Dendrochronologia.

[29] Holmes R.L. (1983). Computer-assisted quality control in tree-ring dating and measurement. Tree Ring Bull..

[30] Burns R.M., Honkala B.H. (1990). Silvics of North America: Vol. 2. Hardwoods.

[31] Bunn A.G. (2008). A dendrochronology program library in R (dplR). Dendrochronologia.

[32] R Development Core Team (2009). R: A Language and Environment for Statistical Computing.

[33] Burnham K.P., Anderson D.R. (2002). Model Selection and Multimodel Inference: A Practical Information-Theoretic Approach.

[34] Liebhold A.M., McCullough D.G., Blackburn L.M., Frankel S.J., von Holle B., Aukema J.E. (2013). A highly aggregated geographical distribution of forest pest invasions in the USA. Divers. Distrib..

[35] Campbell R.W., Sloan R.J. (1977). Forest stand responses to defoliation by the gypsy moth. For. Sci. Monogr..

[36] Gandhi K.J.K., Herms D.A. (2010). Direct and indirect effects of alien insect herbivores on ecological processes and interactions in forests of eastern North America. Biol. Invasions.

[37] Jenkins J.C., Aber J.D., Canham C.D. (1999). Hemlock woolly adelgid impacts on community structure and N cycling rates in eastern hemlock forests. Can. J. For. Res..

[38] Orwig D.A., Cobb R.C., D’Amato A.W., Kizlinski M.L., Foster D.R. (2008). Multi-year ecosystem response to hemlock woolly adelgid infestation in southern New England forests. Can. J. For. Res..

[39] Heichel G.H., Turner N.C. (1983). CO_2_ assimilation of primary and regrowth foliage of red maple (*Acer rubrum* L) and red oak (*Quercus rubra* L): Response to defoliation. Oecologia.

[40] Heichel G.H., Turner N.C. (1984). Branch growth and leaf numbers of red maple (*Acer rubrum* L) and red oak (*Quercus rubra* L): Response to defoliation. Oecologia.

[41] Landhäusser S.M., Lieffers V.J. (2012). Defoliation increases risk of carbon starvation in root systems of mature aspen. Trees Struct. Funct..

[42] Palacio S., Paterson E., Sim A., Hester A.J., Millard P. (2011). Browsing affects intra-ring carbon allocation in species with contrasting wood anatomy. Tree Physiol..

[43] Wareing P.M. (1951). Growth studies in woody species. IV. The initiation of cambial activity in ring-porous species. Physiol. Plant..

[44] Zasada J.C., Zahner R. (1969). Vessel element development in the earlywood of red oak (*Quercus rubra*). Can. J. Bot..

[45] Krause C., Morin H. (1995). Impact of spruce budworm defoliation on the number of latewood tracheids in balsam fir and black spruce. Can. J. For. Res..

[46] Speer J.H., Swetnam T.W., Wickman B.E., Youngblood A. (2001). Changes in pandora moth outbreak dynamics during the past 622 years. Ecology.

[47] Vejpustková M., Holuša J. (2006). Impact of defoliation caused by the sawfly *Cephalcia lariciphila* (Hymenoptera: Pamphilidae) on radial growth of larch (*Larix decidua* Mill.). Eur. J. For. Res..

[48] Fajvan M.A., Rentch J., Gottschalk K. (2008). The effects of thinning and gypsy moth defoliation on wood volume growth in oaks. Trees Struct. Funct..

[49] Mcgraw J.B., Gottschalk K.W., Vavrek M.C., Chester A.L. (1990). Interactive effects of resource availabilities and defoliation on photosynthesis, growth, and mortality of red oak seedlings. Tree Physiol..

[50] Hoogesteger J., Karlsson P.S. (1992). Effects of defoliation on radial stem growth and photosynthesis in the mountain birch (*Betula pubescens* ssp. *tortuosa*). Funct. Ecol..

[51] Simmons M.J. (2013). The Individual Tree and Forest Stand Level Impacts of Winter Moth Defoliation in Eastern Massachusetts, USA.

[52] Naidoo R., Lechowicz M.J. (2001). Effects of gypsy moth on radial growth of deciduous trees. For. Sci..

[53] Kozlowski T.T., Pallardy S.G. (1997). Growth Control in Woody Plants.

[54] Mattson W.J., Herms D.A., Witter J.A., Allen D.C. Woody plant grazing systems: North American outbreak folivores and their host plants. Proceedings of the Joint IUFRO Working Party Symposium.

[55] Davidson C.B., Gottschalk K.W., Johnson J.E. (1999). Tree mortality following defoliation by the European gypsy moth (*Lymantria dispar* L.) in the United States: A review. For. Sci..

[56] Eisenbies M.H., Davidson C., Johnson J., Amateis R., Gottschalk K. (2007). Tree mortality in mixed pine-hardwood stands defoliated by the European gypsy moth (*Lymantria dispar* L.). For. Sci..

[57] Staley J.M. (1965). Decline and mortality of red and scarlet oaks. For. Sci..

[58] Wargo P.M. (1996). Consequences of environmental stress on oak: Predisposition to pathogens. Ann. Sci. For..

[59] Reinikainen M., D’Amato A.W., Fraver S. (2012). Repeated insect outbreaks promote multi-cohort aspen mixedwood forests in northern Minnesota, USA. For. Ecol. Manag..

[60] Mack M.C., D’Antonio C.M. (1998). Impacts of biological invasions on disturbance regimes. Trends Ecol. Evol..

[61] Orwig D.A. (2002). Ecosystem to regional impacts of introduced pests and pathogens: Historical context, questions and issues. J. Biogeogr..

[62] Macphee A.W. (1967). The winter moth, *Operophtera brumata* (Lepidoptera: Geometridae), a new pest attacking apple orchards in Nova Scotia, and its cold hardiness. Can. Entomol..

[63] Medvigy D., Clark K.L., Skowronski N.S., Schafer K.V.R. (2012). Simulated impacts of insect defoliation on forest carbon dynamics. Environ. Res. Lett..

